# Phenolic Extracts from *Acacia mangium* Bark and Their Antioxidant Activities

**DOI:** 10.3390/molecules15053567

**Published:** 2010-05-14

**Authors:** Liangliang Zhang, Jiahong Chen, Yongmei Wang, Dongmei Wu, Man Xu

**Affiliations:** 1 Institute of Chemical Industry of Forest Products, CAF; National Engineering Lab. for Biomass Chemical Utilization; Key and Open Lab. on Forest Chemical Engineering, SFA; Key Lab. of Biomass Energy and Material, Jiangsu Province, Nanjing 210042, China; 2 Institute of New Technology of Forestry, CAF, Beijing 100091, China

**Keywords:** *Acacia mangium*, phenolic compounds, condensed tannins, antioxidant activities, central composite design

## Abstract

Phenolic compounds are present at very high concentrations in the bark of *Acacia mangium*. These compounds are known to have strong antioxidant activity and thus different beneficial effects on human health. Phenolic compounds in bark of *A. mangium* were extracted and their antioxidant activities were investigated using the 1,1-diphenyl-2-picrylhydrazyl (DPPH) free radical-scavenging and ferric-reducing antioxidant power (FRAP) assays. A central composite design has been employed to optimize the experimental conditions for a high total phenolic content and antioxidant activity. The desirability function approach has been employed to simultaneously optimize the three responses: total phenols, antiradical activity and FRAP. An extraction time of 90 min, liquid-solid ratio of 5, and temperature of 50 °C was predicted for the optimum experimental conditions using the desirability function. A significant linear relationship between antioxidant potency, antiradical activity and the content of phenolic compounds of bark extracts was observed. The structures of condensed tannins isolated from *A. mangium* were characterized by MALDI-TOF MS analyses. Condensed tannin oligomers from *A. mangium* were shown to be heterogeneous mixtures consisting of procyanidin and prodelphinidin structural units with polymerization degrees up to 9.

## 1. Introduction

Phenolic compounds, which are secondary metabolites in plants, are known to be responsible for antioxidant effects. Recent epidemiological studies have strongly suggested that consumption of certain plant materials may reduce the risk of chronic diseases related to oxidative stress on account of their antioxidant activity and thus promote general health benefits [1]. On the other hand, in the food industry, antioxidants are used to retard the oxidative degradation of fats by inhibiting the formation of free radicals. Synthetic antioxidants, such as butylated hydroxyanisole (BHA), butylated hydroxytoluene (BHT) and propyl gallate (PG) are widely used; however, the use of synthetic antioxidants in food products is being questioned [2,3]. Consumers have also become more cautious about the nutritional quality and safety of food additives. In response to the growing consumer demand, investigations on antioxidants from natural sources have gained interest [4,5,6,7]. Fruits and vegetables are the main sources of phenolic compounds in the human diet. Other sources, such as bark extracts from high plants, also have received particular attention as sources of antioxidants [8]. Recent antioxidant studies have focused on the improvement of phenol extraction techniques from some natural products [9]. 

*Acacia mangium* Willd. is a tropical Mimosaceae tree species widely planted in Southeast Asia, India and the southern provinces of China for reforestation and soil rehabilitation of degraded land. *A. mangium* is used as raw material for the pulp industry due to its high yield and high-quality fibre [10]. In recent years, it has become a preferred tannin source for the vegetable tannin extract industry in China because of its richness in polyphenolic compounds [11]. However, little information is available concerning the chemical composition and biological activities of *A. mangium*. In particular, polyphenols in *A. mangium* bark have not been well characterized. 

In the present study, the influence of some critical extraction variables on the phenolic yield is researched. An experimental design was performed in order to find the relevant optimal values of these variables. The aim of this study was also to examine the total phenolic content and antioxidant activities in the resultant extracts under different extract conditions. Antioxidant potential has been determined as the free radical scavenging ability using a stable radical, 1,1-diphenyl-2-picrylhydrazyl (DPPH) and ascertained by measuring reducing power. Meanwhile, the condensed tannins from *A. mangium* were separated and the isolated condensed tannins characterized by matrix assisted laser desorption/ionization time-of-flight mass spectrometry (MALDI-TOF MS). 

## 2. Results and Discussion

The Folin-Ciocalteu reagent is popularly used to obtain a crude estimate of the amount of phenolic compounds present in an extract [12,13]. This assay is not specific for particular groups of phenolic compounds, but rather serves to quantify the total concentration of phenolic hydroxyl groups in the plant extract of interest [14,15]. The Folin-Ciocalteu assay for total phenols is affected by several interfering substances such as sulfur dioxide, ascorbic acid, sugar, aromatic amines, organic acid and nonphenolic organic substances that react with the Folin-Ciocalteu reagent [16]. Even with the exclusion of those compounds, different phenolics present different answers with the Folin-Ciocalteu reagent, several flavonoids present low absorption which leads to an underestimation of the various compounds.

Response surface designs permit one to define empirical models (usually quadratic polynomials) that describe accurately how responses behave for all values of the studied variables in the experimental region [17]. The aim of response surface methodology (RSM) is to determine conditions that provide process improvements. 

In order to calculate quadratic regression model coefficients, each design variable has to be studied at three distinct levels at least and, consequently, the central composite design (CCD) is often used to provide estimation of a second-order equation. Among the standard designs applied in RSM, the CCD represents a good choice because of its high efficiency with respect to the number of runs required. The key factors, selected during the optimization process, were: time of contact (*U*_1_), temperature (*U*_2_) and liquid-solid ratio (*U*_3_). The center value and the variation step taken for each variable defined the spherical experimental domain, as reported in Table 1. 

The experimental matrix for three factors (CCD) consists of fifteen experiments, expressed in coded variables *X_i_*, while the corresponding experimental plan, carried out in the laboratory, gives the runs expressed in real variables *U_i_*, as shown in Table 2. The central point was repeated six times to estimate the experimental error variance. All experiments were performed in randomized order to minimize the effects of uncontrolled factors that may introduce a bias on the measurements. Three experimental responses were studied: *Y*_1_ = the content of total phenols; *Y*_2_ = antiradical activity; *Y*_3_ = FRAP value. The experimental results are reported in Table 2.

A classical second-degree model was postulated for each experimental response *Y_i_*, as follows:

*Y_i_* = *β*_0_ + *β*_1_*X*_1_ + *β*_2_*X*_2_ + *β*_3_*X*_3_ + *β*_11_*X*_1_^2^ +*β*_22_*X*_2_^2^ + *β*_33_*X*_3_^2^ + *β*_12_*X*_1_*X*_2_ + *β*_13_*X*_1_*X*_3_ + *β*_23_*X*_2_*X*_3_

where *X*_1_, *X*_2_, *X*_3_, are the coded variables, *β_i_* represent the model coefficients. To obtain a simple and yet a realistic model, the insignificant terms (Prob > *F*′ > 0.05) are eliminated from the model through a “backward elimination” process. The statistical parameters obtained from the ANOVA for the reduced models are given in Table 3. For all the reduced models, Prob > *F*′ < 0.05 are obtained, implying that these models are significant. The adequate precision value is a measure of the “signal (response) to noise (deviation) ratio”. A ratio greater than four is desirable [17,18]. In this study, the ratio was found to be around 10, which indicates an adequate signal and therefore the model is significant for the extracting process. As can be seen in the Table 3, no interaction between factors is statistically significant for three responses. 

**Figure 1:**
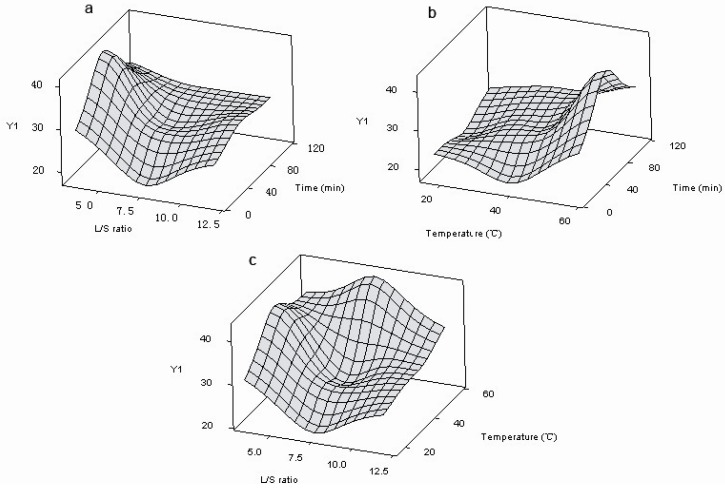
Three-dimensional plot of total phenolic content (*Y*_1_). (a) Response plot of contact time (*U*_1_) *vs.* L/S ratio (*U*_3_); (b) Response plot of contact time (*U*_1_) *vs.* temperature (*U*_2_); (c) Response plot of temperature (*U*_2_) *vs.* L/S ratio (*U*_3_).

The surface response graphs were drawn by plotting the response variation against two of the factors, while the third is held constant at a specified level, usually the center value. It can be observed that the response antiradical activity (*Y*_2_) and FRAP value (*Y*_3_) show the same behavior in the studied experiment domain. For this reason, only the response surfaces for content of total phenols (*Y*_1_), considering all possible variable interactions, are reported in Figures 1a-c. The areas of interest for the maximization of the total phenolic content are examined. As reported in Figure 1, an increase of liquid-solid ratio (*U*_3_) results in a decrease of total phenolic content (*Y*_1_), while the contact time (*U*_1_) has no important effect in the studied domain on the considered response. An increase of temperature (*U*_2_) results in an increase of total phenolic content (*Y*_1_).

The desirability function approach to multiresponse optimization is a useful technique for the analysis of experiments in which several responses have to be optimized simultaneously [19]. The measured properties of each response *Y_i_*, *i* = 1, 2, …*m*, are transformed to a dimensionless desirability scale (*d_i_*), defined as *partial desirability function*. This makes it possible to combine results obtained for properties measured on different scales.

The scale of the desirability function ranges between *d* = 0, for a completely undesirable response, and *d* = 1, if the response is at the target value. The responses total phenols, inhibition percentage and FRAP were transformed into an appropriate desirability scale *d*_1_, *d*_2_ and *d*_3._ Once the function *d_i_* is defined for each of the *m* responses of interest, an overall objective function (*D*), representing the *global desirability function*, is calculated by determining the geometric mean of the individual desirabilities. Therefore, the function *D* over the experimental domain is calculated, as follows:

$$D={\left(\prod_{i=1}^{m}d_{i}\right)}^{1/m}$$

Taking into account all requirements for *m* responses, we can choose the conditions on the design variables that maximize *D*. A value of *D* different to zero implies that all responses are in a desirable range simultaneously and consequently, for a value of *D* close to 1, the combination of the different criteria is globally optimal, so as the response values are near target values. 

After calculation by Design-Expert software, an optimal extraction condition with an extraction time of 90 min, liquid-solid ratio of 5, temperature of 50 °C was predicted using the desirability function. The optimal conditions were obtained with a global degree of satisfaction of *D* for the three responses equal to 0.974. 

**Figure 2:**
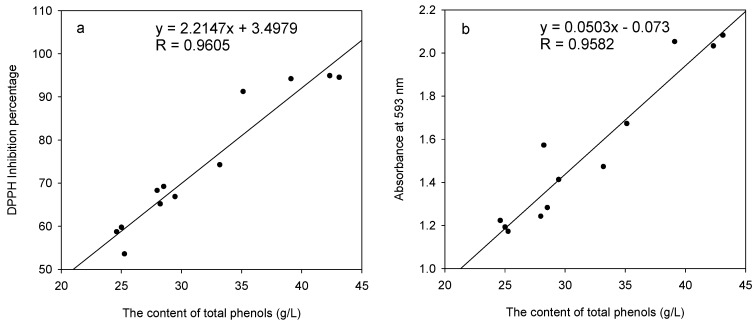
Relationships between the total phenolic content and the free radical scavenging activity (a); the total phenolic content and the ferric-reducing power (b) of the 50% ethanol-water extracts of *A. mangium* bark.

The correlations between the total phenolic content of each fraction and the free radical scavenging activity and reducing power were shown in Figure 2. The free radical scavenging activity and reducing power of the 50% ethanol-water extracts were significantly related to their total phenolic content (*R*_DPPH_ = 0.9605, *R*_FRAP_ = 0.9582) (Figure 2). The 50% ethanol-water extract exhibited the highest radical scavenging activity and ferric-reducing power with the greatest amount of phenolic content. The presence of polyphenolic compounds in extracts of *A. mangium* bark might be responsible for this high antioxidant activity. In addition, the good correlation was observed between the DPPH and FRAP assay, with the regression equation was *y* = 0.0211*x* – 0.0336 (*R* = 0.9272). The FRAP assay was found to be more sensitive than the DPPH free radical-scavenging assay, which is in agreement with the observation of Zheng and Wang [20].

**Figure 3:**
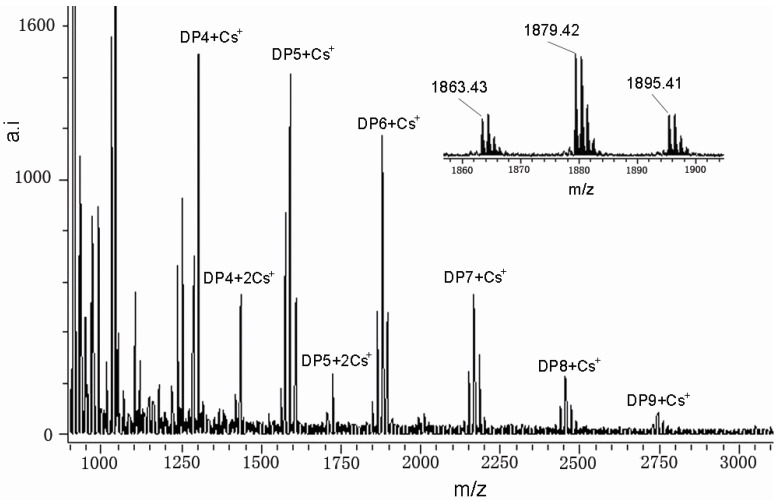
MALDI-TOF positive reflectron mode mass spectra of the condensed tannins from *A. mangium*. Inset is an enlarged spectrum of masses representing hexamer.

Figure 3 shows the MALDI-TOF mass spectra of the condensed tannins isolated from *A. mangium* bark, recorded as Cs^+^ adducts in the positive ion reflectron mode and showing a series of repeating procyanidin polymers. The polymeric character is reflected by the periodic peak series representing different chain lengths. The masses of the highest peaks among the polyflavonoid tannin polymers from *A. mangium* with identical DP increased at the distance of 288 Da, corresponding to one catechin/epicatechin monomer (Table 4). Therefore, prolongation of condensed tannins is due to the addition of catechin/epicatechin monomers. Condensed tannins with a DP of 4 to 9 (*m/z* 1287.27~2727.70) were detected in *A. mangium*. The spectra did not contain ions 2 Da lower than that of the highest peaks among the polyflavan-3-ols polymers, so A-type interflavan ether linkage does not occur between adjacent flavan-3-ol subunits.

In addition to the predicted homopolyflavan-3-ol mass series mentioned above, each DP had a subset of masses 16 and 32 Da higher (Figure 3 and Table 4). These masses can be explained by heteropolymers of repeating flavan-3-ol units containing an additional hydroxyl group (∆16 Da) at the position 5' of the B-ring [21]. Given the absolute masses corresponding to each peak, it was further suggested that they contain procyanidins and prodelphinidins. The galloyl group which showed the mass signals at a distance of 152 Da was not detected in the mass spectra although it was widely distributed in the plant kingdom [22,23]. For the first time, compositional analysis of condensed tannins from *A. mangium* using MALDI-TOF MS has been successfully demonstrated. 

The characterization of the condensed tannins by MALDI-TOF MS analyses was limited with respect to the detection of high mass condensed tannins and by the fact that peak intensities were not related to their concentration in the sample. The quality of the spectrum depended upon parameters such as the tannin/matrix ratio, the target area of the laser, and the crystallization of the mixture on the target. Therefore, it was very difficult to obtain a reproducible spectrum. The main problem is to find a new technique that permits the complete characterization of condensed tannins up to 4,000 Da, which allows an equal sensitivity for both low and high mass polymers.

## 3. Experimental

### 3.1. Sample preparation and chemicals

*A. mangium* bark was supplied by Guangxi Baise Forest Chemicals General Plant, Baise China, and dried at room temperature for two week. It was ground in a knife mill and the powdered bark was sieved to select particles smaller than 1 mm. 1,1-Diphenyl-2-picrylhydrazyl (DPPH) and 2,4,6-tripyridyl-*S*-triazine (TPTZ) were purchased from Sigma Chemical Co. (St. Louis, MO, USA). Reagents and solvents were of analytical grade. Deionized water was used throughout.

### 3.2. Experimental design

Experimental design, data analysis and desirability function calculations were performed by using Design-Expert software version 7.1.3.

### 3.3. Solvent extraction

Twenty five mL capped flasks were used to extract 1 g of ground *A. mangium* bark. Ethanol-water (1:1, v/v) was used as a solvent. Solids were separated by filtration and the crude extracts were analysed for polyphenols and antioxidant activity. The condensed tannins from *A. mangium* bark were extracted and purified as described by Zhang *et al*. [24]. The condensed tannins were freeze-dried and stored at −20 °C before analysis by MALDI-TOF mass spectrometry.

### 3.4. Determination of total phenolics

The content of phenolic compounds in the bark extracts was determined according to the method of Jayaprakasha *et al*. [25]. The extracts were dissolved in water. Aliquots samples (0.5 mL) were mixed with 10-fold-diluted Folin-Ciocalteu reagent (2.5 mL) and 7.5% sodium carbonate (2 mL). The mixture was allowed to stand for 30 min at room temperature before the absorbance was measured spectrophotometrically at 760 nm. A mixture of water and reagents was used as a blank. The final results were expressed as gallic acid equivalents.

### 3.5. Antiradical activity

A DPPH radical-scavenging assay was performed, using the method described by Braca *et al*. [26] to determine the hydrogen-donating ability of the bark extract. Each extract (0.1 mL) in methanol was mixed with methanol solution (3 mL) containing DPPH radicals (0.004%, w/w). The mixture was shaken vigorously and left to stand for 30 min in the dark before measuring the absorbance at 517 nm against a blank. Then the inhibition percentage (IP) of the DPPH radical was calculated using the following equation: IP = [(∆A_517 of control_ − ∆A_517 of sample_)/∆A_517 of control_] × 100. Three replicates were carried out.

### 3.6. Ferric-reducing antioxidant power (FRAP) assay

The ferric-reducing antioxidant power of the extracts was estimated according to the method described by Benzie and Strain [27]. Three mL of FRAP reagent, prepared freshly, was mixed with test sample (0.1 mL), or methanol (for the reagent blank). The FRAP reagent contained 10 mM TPTZ solution (2.5 mL) in 40 mM HCl plus 20 mM FeCl_3_ (2.5 mL) and 0.3 M acetate buffer (pH 3.6, 25 mL). The absorbance of reaction mixture was measured spectrophotometrically at 593 nm after incubation at 25 °C for 5 min. FRAP assay records the change in absorbance at 593 nm owing to the formation of a blue colored Fe(II)-tripyridyltriazine compound from colorless oxidized Fe(III) form by the action of electron donating antioxidants. All solutions were used on the day of preparation.

### 3.7. MALDI-TOF MS analysis 

The MALDI-TOF mass spectra were recorded on a Biflex Ⅲ MALDI-TOF mass spectrometer (Bruker-Daltonics) equipped with a 337 nm nitrogen laser, in reflector mode. The spectra of condensed tannins were obtained from a sum of 100–150 shots and calibrated using angiotensin II (1,046.5 MW), bombesin (1,619.8 MW), ACTHclip18–39 (2,465.2 MW), and somatostatin 28 (3,147.47 MW) as external standards. Cesium chloride and 2,5-dihydroxybenzoic acid (DHB) as the matrix were used to enhance ion formation. Amberlite IRP-64 cation-exchange resin (Sigma-Aldrich), equilibrated in deionized water, was used to deionize the analyte/matrix solution thrice. An aqueous solution of cesium chloride (0.5 µL, 1 mg/mL) was added to the sample solution (1.5 µL, 10 mg/mL aqueous) followed by addition of an equal volume of DHB (10 mg/mL aqueous solution). The resulting solution was placed (1.0 µL) onto the MALDI plate, evaporated and introduced into the mass spectrometer [28]. 

## 4. Conclusions

*A. mangium* bark contains considerable quantities of antioxidant phenols. This is of great importance for the industry, since the extracts of these byproducts are finding increasing applications as active substances for cosmetic and pharmaceutical compositions. Industrially, however, the economic feasibility of the extraction process involves a search for optimum extraction conditions, in order to maximize process efficiency. This paper shows how an experimental design approach led us to obtain an effective extraction of total phenolic compounds with respect of a reduced number of experiments. By means of RSM and multiresponse optimization, the three considered responses were modeled in the experimental domain with a good fitness. In addition, the use of an appropriate chemometric methodology during optimization study has given an indication of method robustness. Compared to empirical methods, chemometrics can greatly simplify the optimization of extraction process finding the appropriate experimental conditions. Structures of the condensed tannins from *A. mangium* were characterized by MALDI-TOF MS analyses and it was shown that the condensed tannins consisted of both procyanidins and prodelphinidins with polymerization degrees up to 9.

## Figures and Tables

**Table 1:** Experimental domain for the three studied factors.

Factors	Center	Variation step
*U*_1_: time of contact (min)	60	30
*U*_2_: temperature (℃)	37.5	12.5
*U*_3_: liquid-solid ratio	7.5	2.5

**Table 2:** Experimental design and results of the central composite design.

No.	Coded variables				Real variables				Responses		
	*X*_1_	*X*_2_	*X*_3_		*U*_1_	*U*_2_	*U*_3_		*Y*_1_	*Y*_2_	*Y*_3_
1	–1	–1	–1		30	25	5		35.17	91.08	1.67
2	1	–1	–1		90	25	5		42.37	94.77	2.03
3	–1	1	–1		30	50	5		43.16	94.40	2.08
4	1	1	–1		90	50	5		39.15	94.06	2.05
5	–1	–1	1		30	25	10		28.57	69.06	1.28
6	1	–1	1		90	25	10		29.51	69.74	1.41
7	–1	1	1		30	50	10		28.02	68.16	1.24
8	1	1	1		90	50	10		33.23	74.13	1.47
9	–1.6818	0	0		9.5	37.5	7.5		18.30	48.32	0.86
10	1.6818	0	0		110.5	37.5	7.5		28.21	68.34	1.31
11	0	–1.6818	0		60.0	16.5	7.5		20.93	50.78	0.93
12	0	1.6818	0		60.0	58.5	7.5		42.87	94.60	2.07
13	0	0	–1.6818		60.0	37.5	3.3		40.65	94.27	2.02
14	0	0	1.6818		60.0	37.5	11.7		29.64	67.01	1.25
15	0	0	0		60.0	37.5	7.5		24.66	55.57	1.20
16	0	0	0		60.0	37.5	7.5		26.28	55.06	1.19
17	0	0	0		60.0	37.5	7.5		25.06	53.61	1.19
18	0	0	0		60.0	37.5	7.5		25.31	53.45	1.17
19	0	0	0		60.0	37.5	7.5		26.42	55.62	1.20
20	0	0	0		60.0	37.5	7.5		24.86	55.08	1.21

The relation *U_i_* = *U_i_*^0^ + *X_i_*∆*U_i_* allows one to switch from coded variables to real variables. *U_i_*^0^, value of real variable, *i*, at the center of the experimental domain; ∆*U_i_*, variation step of the real variable, *i*, for a unit variation of the coded variable *X_i_*; *Y*_1,_ the content of total phenols, was expressed as gallic acid equivalents; *Y*_2,_ antiradical activity of extracts, was expressed as inhibition percentage; *Y*_3,_ FRAP value, was expressed as absorbance at 593 nm. Antioxidant activities were determined with 100-fold-diluted extracts

**Table 3:** Reduced response models and statistical parameters obtained from ANOVA (after backward elimination).

Response	Reduced response models*^a^*	Adjusted *R*^2^	Model *P* value	% CV	Adequate precision
*Y*_1_	25.44 + 3.28*X*_2_ – 4.32*X*_3_ + 3.22*X*_2_^2^ + 4.37*X*_3_^2^	0.742	0.000	14.05	9.130
*Y*_2_	57.23 + 5.84*X*_2_ – 10.18*X*_3_ + 8.20*X*_2_^2^ + 11.01*X*_3_^2^	0.774	0.000	13.18	10.411
*Y*_3_	1.19 + 0.11*X*_1_ + 0.17*X*_2_ – 0.27*X*_3_ + 0.16*X*_2_^2^ + 0.20*X*_3_^2^	0.823	0.000	13.50	11.519

***^a^*** Only significant coefficients with *P* < 0.05 are included. Factors are in coded levels.

**Table 4:** Observed and calculated masses of heteropolyflavan-3-ols by MALDI-TOF MS.

Polymer	Number of catechin unit	Number of gallocatechin unit	Calculated [M + Cs]^+^	Observed [M + Cs]^+^
Tetramer	4	0	1287	1287.27
	3	1	1303	1303.26
Pentamer	5	0	1575	1575.34
	4	1	1591	1591.34
	3	2	1607	1607.33
Hexamer	6	0	1863	1863.43
	5	1	1879	1879.42
	4	2	1895	1895.41
Heptamer	7	0	2151	2151.52
	6	1	2167	2167.51
	5	2	2183	2183.49
Octamer	8	0	2439	2439.56
	7	1	2455	2455.61
	6	2	2471	2471.68
Nonamer	9	0	2727	2727.70
	8	1	2743	2743.64
	7	2	2759	2760.65
